# The Effectiveness of Peyton’s 4-Step Approach to Teach Resuscitation Skills: A Randomized Controlled Clarification Study

**DOI:** 10.1177/23821205251358090

**Published:** 2025-07-24

**Authors:** Denisa Cenaj, Leonie Schulte-Uentrop, Leonie Fee Laura Kröger, Josephine Küllmei, Jan-Marcus Haus, Parisa Moll-Khosrawi

**Affiliations:** 1 Department of Anesthesia, 37734 University Medical Center Hamburg-Eppendorf, Hamburg, Germany

**Keywords:** curriculum development, simulation-based medical education, cardiopulmonary resuscitation, nontechnical skills, technical skills, procedural skills, learning process, Peyton’s 4-step approach

## Abstract

**Objectives:**

The aim of this study was to compare the effectiveness of the Peyton approach with the traditional 2-step approach instruction and to clarify if a possible superiority can be attributed to specific skill domains. The primary and secondary outcome were the quality of resuscitation, reflected by technical (TS)- and nontechnical skills (NTS). The tertiary outcome were the subjective learning gains of the undergraduates.

**Methods:**

In a randomized controlled simulation study, second year medical undergraduates participated in compulsory Advanced Cardiac Life Support training (ACLS). The control group received the 2-step approach and the intervention group Peyton’s 4 step approach as a training instruction.

**Results:**

N = 290 second year medical undergraduates participated in the study. There were no relevant differences between both groups in purely haptically skills like chest compression. The intervention group showed significantly better procedural skills of ACLS skill domains, resulting in lower no-flow time during the scenarios (*t*(120) = 2.132, *P* = .035)). NTS of both groups did not differ (*t*(150) = 1.694, *P* = .092)). Undergraduates of the intervention group reported significant higher learning gains for procedural ACLS skills, like performing the algorithm (*P* < .001).

**Conclusion:**

Integrating Peyton's 4-step approach into structured ACLS training enhances procedural cardiopulmonary resuscitation skills, adherence to the ACLS algorithm, and skill retention in advanced stages of medical education, especially when undergraduates have prior resuscitation experience. While the method shows limited benefit for teaching discrete tactile skills in earlier stages, its strategic inclusion in later phases can optimize curriculum design by aligning advanced teaching methods with learners’ developmental needs. These findings highlight the importance of tailoring ACLS education to maximize training effectiveness and improve resuscitation outcomes.

## Introduction

Sudden cardiac arrest (SCA) is one of the leading causes of mortality worldwide.^1,2^ It is defined as the cessation of cardiac activity in association with the absence of systemic circulation.^3,4^ The provision of high-quality cardiopulmonary resuscitation (CPR), ^5,6^ and the early use of an automated external defibrillator (AED)^7^ substantially enhance both, survival rates and long-term outcomes^2,8^ of individuals suffering SCA.^9,10^ A short “no-flow-time”—the period during which no blood flow occurs—is essential for enhancing the neurological outcomes of SCA survivors.^11-14^ Therefore, it is of particular importance that healthcare professionals receive comprehensive training in CPR skills^12^ and CPR trainings should be early incorporated into the curricula of medical and nursing schools, to provide future healthcare providers with the requisite knowledge and abilities to deliver Basic- and Advanced Cardiac Life Support (BLS, ACLS). These trainings should cover technical (TS) and nontechnical skills (NTS).^15-17^ NTS are necessary to complement TS, ensuring high-quality CPR and enhancing patient safety.^17,18^

Simulation-based medical education (SBME) has been identified as the appropriate didactic method to train CPR skills.^19^ However, the optimal instructional concept for SBME remains unclear. As skill instruction has an impact on future performance, a variety of methods have been described in this regard.^19-21^ Among these is the stepwise approach, first introduced by Walker and Peyton in 1998.^22^ Peyton's 4-steps approach is firmly grounded in educational theory and has gained notable popularity in recent years for the instruction of technical skills in healthcare education.^23^ It comprises the following 4 steps:^23^

- **Step 1—Demonstrate:** In this initial phase, the trainer demonstrates the skill at a normal pace, providing no additional commentary.
- **Step 2—Talk the trainee through:** In the second step, the trainer once again demonstrates the skill while offering detailed descriptions of each substep involved.
- **Step 3—Trainee talks trainer through:** Next, the trainer performs the skill for a third time, based on the substeps described to them by the trainee.
- **Step 4—Trainee performs:** Finally, in the fourth step, the trainee takes the lead and independently performs the skill.

Several studies have explored the efficacy of the Peyton approach in the context of medical education. On the one hand, favorable outcomes have been described for mainly complex skill acquisition, NTS like professionalism, and doctor-patient communication.^24-27^ On the other hand, a few studies have indicated that the Peyton approach is not more effective than other approaches.^28-30^

In summary, there is limited evidence supporting the superiority of Peyton's approach over other teaching methods.^31^ Although it has been adopted by several resuscitation councils, its effectiveness in teaching CPR skills remains also inconclusive, highlighting the need for further research to identify optimal CPR training practices.^32^

Therefore, the aim of this randomized controlled simulation study was to investigate the acquisition of ACLS skills among medical undergraduate students, comparing the efficacy of the 4-stage Peyton instructional approach with the traditional 2-stage approach. The primary outcome of the study was the acquisition of TS, while the secondary outcome focused on the development of NTS. The outcomes were assessed through performance in an end-of-course ACLS scenario test. Additionally, the tertiary outcome evaluated the subjective learning gains reported by the participating undergraduates.

## Methods

The reporting of this study conforms to the CONSORT 2025 statement (supplemental file 1).^33^

### Study Design

This prospective, randomized cohort simulation study was conducted at the Department of Anesthesiology, Medical University Center Hamburg-Eppendorf, during the winter and summer semesters of 2022/23.

### Study Setting and Participants

At the Medical University Center of Hamburg, the undergraduate curriculum (iMED) is vertically integrated and organized as learning spirals.^34^ During the development of iMED, emergency medicine competencies had been emphasized as crucial and therefore, the incorporation of CPR in education begins early, with BLS training in the first year of medical school, progressing to fundamental aspects of emergency medicine (begin of second year), ACLS in the end of the second year and further trainings that gain complexity in the third and fourth year.

The trainings are compulsory and organized within learning modules of the curriculum in each semester. The study was conducted within the ACLS training of second year undergraduates.

All undergraduate medical students attending the simulation training during the study period were eligible for participation.

Prior to each semester, the dean's office organizes schedules in which students are divided into small groups for each teaching unit, with a maximum of 19 students participating in each ACLS training session.

### Study Procedure: ACLS Training and Intervention

The ACLS training is designed with predetermined learning objectives, which are accessible through the university's online platform. Each training is composed of a theoretical/demonstration and a simulation unit: beginning with a 45min seminar, in which fundamentals of BLS are reviewed and then an introduction and in-depth look at ACLS is given, followed by a demonstration of a ACLS scenario. The simulation unit includes 3 standardized simulation scenarios of cardiac arrest. The undergraduates are divided in smaller groups (5-7 undergraduates per subgroup) to rotate through the scenarios, which are conducted in different rooms of the simulation center of the Department of Anesthesiology, using high fidelity manikins (Resusci Anne, Laerdal Medical AS, Stavanger, Norway).

Every scenario is actively conducted by 3 undergraduates of the smaller groups who are randomly selected to partake the roles of an emergency physician (team leader) and paramedics/nurses. The remaining students in the smaller subgroup assume the role of neutral observers. Each group is closely supervised by an instructor, who is an experienced anesthesiologist and medical educator. In each scenario, instructors refrain from intervening or speaking, even in cases where students are not responding appropriately. Following each scenario, a conventional teacher-led debriefing is conducted, with a focus on both TS and NTS.

The structure of both training groups (intervention and control) was identical and standardized, regarding the learning objectives and scenarios. The training of the control group was carried out in accordance with the curriculum. The intervention took place during the demonstration phase at the end of the theoretical unit in the large group setting.

To prevent bias caused by technical application problems, we made sure that all instructors were sufficiently trained in advance regarding the use of the evaluation forms and high-fidelity manikins.

### Intervention

Each group was randomly assigned (using simple computerized random numbers) to either an intervention (IC)- or control group (CG). Randomization was conducted by one of the principal investigators (PM-K). The medical educators were not blinded, the undergraduates were blinded.

Prior to their simulation unit, the CG received a demonstration of an ACLS scenario with simultaneous explanations, conducted by the instructors. The IG received a demonstration based on Peyton's 4-step approach, which was also executed by the instructors. First, the scenario was demonstrated without explanations (step 1). Then, the demonstration included detailed descriptions of each substep (step 2). Following this, the scenario was performed, with the undergraduates describing the substeps to the instructors (step 3). The fourth step (the trainee performs) was then conducted in the smaller groups within the simulation unit.

### Data Collection and Assessment Tools

#### Primary and Secondary Outcome: Quality of CPR-TS and NTS

TS and NTS were assessed for each simulated scenario, using validated assessment tools, as well as mannequin CPR reports.

TS were rated with the “Graham-Score” (adapted from Graham and Lewis, 2000)^35^ (1 score per scenario/team). This assessment tool has originally been developed to assess the quality of BLS.^35^ In a pilot study we have expanded and adapted the scoring for ACLS. It is composed of a total of 12 items on which penalty points are given regarding TS, reflecting incorrect performance of each ACLS component. The best possible ACLS performance is combined with 0 penalty points, the worst performance with 200 penalty points.

Alongside the application of the Graham scoring, CPR performance metrics were extracted from the mannequin. These data, recorded using a Laerdal CPR meter, provide detailed insights into resuscitation quality, such as compression depth, rate, recoil, and other performance indicators essential for evaluating adherence to CPR guidelines.

NTS performance of the team leader (the undergraduate taking the role of the physician), was assessed using the German version of “Anaesthesiology students’ Non-Technical skills” (AS-NTS).^36^ AS-NTS has been developed and broadly validated for direct assessment of NTS directly during undergraduate simulation training.

NTS are rated on 3 dimensions separately using a 5-point Likert scale (1 = “very good”; 5 = “very poor”). Examples of corresponding very good or poor performances in the respective of each dimension are provided on the AS-NTS.^36^

The 3 dimensions are:

- “Task planning, prioritization, and problem-solving”;
- “Teamwork and leadership”; and
- “Team orientation”.

#### Tertiary Outcome: Subjective Learning Gain of the Students

The tertiary outcome was the learning gain which was assessed by the comparative student self-assessment (CSA) tool.^37^ The CSA is validated tool to appraise undergraduate medical curricula at the level of specific learning objectives. For our study we have adapted the items for learning objectives addressing ACLS by a total of 14 items. Each item is rated on a 6-point Likert scale (1 = “mostly applies”; 6 = “does not apply”). The CSA questionnaires were administered once before the seminar and again at the conclusion of the practical training session.

### Statistical Analysis

Statistical analysis was performed using IBM SPSS Statistics Version 29.0.2.0.

Descriptive statistics were used for the calculation of mean values of the outcome measures (AS-NTS dimensions, Graham score criterions, CSA elements).

For each analysis, the 2-stage *P*-value was determined at a significance level of 0.05.

An unpaired t-test was conducted to analyze the differences between groups regarding TS and NTS. Normal distribution of the data and homogeneity of error variances in each group, as assessed by Levene’s test (*P* > .05) was given.

For the tertiary outcome (CSA), the disparities between pretraining and post-training sessions were compared by conducting score subtractions: CSA gain (points)= CSA_pre_ – CSA_post_. Participants who rated themselves with the highest possible score (1 = “mostly applies”) at the pretraining self-assessment were excluded from the analysis, since no learning gain can be observed in relation to these points.

The data analysis was carried out in a pseudonymized and anonymized manner. The data is stored on computers that are not connected to the internet and are only accessible to the principal study investors.

## Results

### Participants

**Figure 1.**
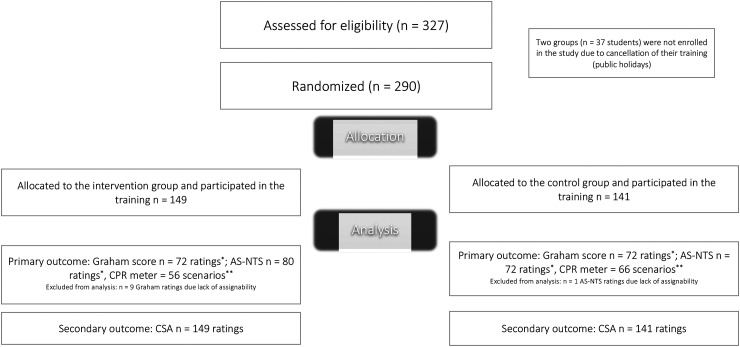
Participant Flow of the Study. *Subgroups of Undergraduates Simulated the Scenarios and the AS-NTS and the Graham Score were Assessed for Each Scenario, Therefore the Number of Assessments is Smaller than the Total Number of Analysed Students. **Data from the Integrated CPR Manikin Meter was Lost Due to Technical Issues Known to the Company, Such as Transmission Errors or Device Crashes, Which Prevented Data Extraction. Abbreviations: AS-NTS, Anesthesiology Students’ Nontechnical Skills; CPR, Cardiopulmonary Resuscitation; CSA, Comparative Self-Assessment.

A total of 326 undergraduates were assessed for eligibility and 290 undergraduates (n = 149 IG-, n = 141 CG) were enrolled within the study and the final analysis. Thirty-seven undergraduates were excluded, as their training was canceled due to public holidays. Figure 1 depicts the participant flow as well as the assessment of the endpoints.

As shown in Table 1, the demographics of the randomized and analyzed students were not significantly different.

**Table 1.** Demographic Data of the Study Participants Included in the Final Analysis.

	Intervention Group (n = 149)	Control Group (n = 141)	*P*
Age (mean), years	20.4	20.9	> .05
Gender, n (%)			
Female	80 (53.7)	76 (53.9)	> .05
Male	69 (46.3)	65 (46.1)	> .05
Additional CPR-and or/ emergency training. (Despise the trainings of the curriculum) Prior medical knowledge or medical work experience (training as paramedic nurse etc), n (%)	2 (1.3)	1 (0.71)	> .05

Abbreviation: CPR, cardiopulmonary resuscitation.

### Primary Outcome: Quality of CPR, TS

Compared to the CG, the IG showed overall better TS as assessed by the Graham score, with a mean difference of 8.056 penalty points, *t*(142)=−1.994, *P* = .048. The subscorings of the Graham score are depicted in Table 2. As shown, there were no significant differences between the groups regarding haptically skills (frequency, depth, or recoil of chest compressions). However, the IG received significantly lower penalty points regarding “inform others/call for help” and “circulation control.”

**Table 2.** Penalty Points of Both Groups Assessed by the Adapted Graham Scoring.

Graham Score			Intervention Group (n = 72 Scorings)		Control Group (n = 72 Scorings)		Differences Between the Groups				
Subscoring	Value	Penalty Points	*M*	*SD*	*M*	*SD*	*MD*	*T(df)*	*95% CI* *LL*	*95% CI* *UL*	*P*
*Category 1: checking for consciousness*	Right /done	0	.07	.59	.14	.83	−.069	−.580 (142)	−.306	.167	.563
	Wrong / not done	5									
*Category 2: inform others / call for help*	Right / done	0	.00	.00	.49	1.50	−.493	−2.787 (141)	−.843	−.143	.006
	Wrong / not done	5									
*Category 3: Open Airway*	Right	0	1.53	4.33	2.96	5.95	−1.430	−1.645 (141)	−3.149	.289	.102
	Inadequate	10									
	No attempt / wrong	20									
*Category 4: assess breathing*	Right / done	0	.42	1.39	.90	2.82	−.486	−1.310 (142)	−1.220	.247	.192
	Inadequate	5									
	Wrong / not done	20									
*Category 5: circulation control*	Right / done	0	1.39	4.21	4.58	7.54	−3.194	−3.138 (142)	−5.207	−1.182	.002
	Inadequate	5									
	Wrong / not done	20									
*Category 6: (CC) hand position*	Right	0	3.24	5.55	2.54	4.99	.704	.795 (140)	−1.047	2.455	.428
	Wrong	10									
	Grossly wrong	20									
*Category 7: (CC) frequency*	100-120/min	0	2.08	3.22	3.13	3.79	−1.042	−1.776 (142)	−2.201	.118	.078
	120-140/min or 80-100/min	5									
	>140/min or <80/min	15									
*Category 8: (CC) depth*	Right (5-6 cm)	0	4.86	5.31	5.42	5.55	−.556	−.614 (142)	−2.344	1.233	.540
	Too light (3-5 cm), too deep (>6 cm)	10									
	Wrong (<3 cm) or no compression	20									
*Category 9: (CC) chest recoil*	Right	0	2.85	3.23	2.64	3.27	.204	.375 (140)	−.874	1.282	.708
	Slightly incomplete (<2 cm)	5									
	Wrong (no recoil or >2 cm)	15									
*Category 10: ventilation*	Adequate (>400 mL)	0	5.83	5.50	5.83	6.87	.000	.000 (142)	−2.050	2.050	1.00
	Inadequate (<400 mL)	10									
	No or wrong (<200 mL)	20									
*Category 11: use and timing of defibrillation*	Right/ used	0	.83	4.03	.56	3.31	.278	.450 (142)	−.936	1.492	.625
	Wrong/ not used	20									
*Category 12: drug administration (adrenaline)*	Right timing	0	3.24	5.29	5.00	5.31	−1.761	−1.988 (141)	−3.512	−1.009	.049
	Wrong timing	10									
	No preparation / not done	20									
*Mean overall score*			*25*.*97*	*21*.*67*	*34*.*03*	*26*.*56*	*−8*.*056*	*−1.994 *(*142)*	*−16*.*041*	*−*.*070*	.*048*

Abbreviations: CC, chest compressions; CI, confidence interval; df, degrees of freedom; LL, lower limit; MD, mean difference; MV, mean value; SD, standard deviation; T, t-distribution; UL, upper limit.

The CPR metrics showed a significantly lower no flow time in the IG, reflected by “Flow-time percentage,” with a mean difference of 7.437%, *t*(120)= 2.132, *P* = .035.

“Average number of compressions per cycle” (mean difference 9.791 compressions, *t*(119)= 3.212, *P* = .02) and “First time to compression” (mean difference 15.514 s, *t*(120)= −2.61, *P* = .02; Supplemental file 2). There were no significant differences in haptically skills, only the release depth was better in the intervention group (mean difference 9.379 mm *t*(120)= 2.307, *P* = .020).

### Secondary Outcome: Quality of CPR, NTS

Both groups performed good to average on the 3 NTS dimensions. There was no statistically significant difference between both groups (Table 3).

**Table 3.** NTS Performance Assessed With the AS-NTS (Group Comparison).

AS-NTS Sub Scoring	Intervention Group (n = 80 Scorings)		Control Group (n = 72 Scorings)		Differences Between the Groups				
	*M*	*SD*	*M*	*SD*	*MD*	*T(df)*	*95% CI* *LL*	*95% CI* *UL*	*P*
D1	2.35	1.020	2.13	0.948	.225	1.404 (150)	−.092	.542	.163
D2	2.36	1.070	2.11	0.912	.251	1.550 (150)	−.069	.572	.123
D3	2.43	1.065	2.17	.839	.258	1.649 (150)	−.051	.568	.101
*Mean overall score*	*2*.*38*	.*973*	*2*.*13*	.*786*	.*245*	*1.694 *(*150)*	*−*.*041*	.*531*	.*092*

Abbreviations: AS-NTS, anesthesiology students’ nontechnical skills; CI, confidence interval; D1, dimension 1 of AS-NTS “Planning tasks, prioritizing and problem-solving”; D2, dimension 2 of AS-NTS “Teamwork and leadership”; D3, dimension 3 of AS-NTS “Team orientation”; LL, lower limit; M, mean value; S, sum scoring of AS-NTS; SD, standard deviation; UL, upper limit.

### Tertiary Outcome: Subjective Learning Gain

The undergraduates of both groups reported for each dimension of the CSA significant learning gains. The group comparison showed that the undergraduates of the intervention group reported significant higher learning gains on dimensions targeting mainly procedural competencies of ACLS (Table 4).

**Table 4.** Subjective Learning Gains of Undergraduates (Group Comparison).

Items		Intervention Group	Adjusted *P*-Value	CSA-Values Pretraining in Points	CSA-Values Post-Training in Points	Mean Learning Gain Points (Pre-Post)
		Control Group				
1	I feel capable of describing the algorithm for Basic Life Support and Advanced Life Support	Intervention (n = 149)	.002	2.85	1.47	1.376
		Control (n = 141)		2.56	1.70	.858
2	I feel capable of performing the Basic Life Support algorithm	Intervention (n = 149)	.115	2.41	1.44	.973
		Control (n = 141)		2.49	1.57	.922
3	I feel capable of performing the Advanced Life Support algorithm	Intervention (n = 149)	.008	3.88	1.75	2.132
		Control (n = 141)		3.71	1.99	1.727
4	I feel capable of recognizing irregular breathing	Intervention (n = 149)	.008	2.30	1.82	.476
		Control (n = 141)		2.72	2.09	.638
5	I feel capable of recognizing/identifying cardiac arrest	Intervention (n = 149)	<.001	2.11	1.39	.718
		Control (n = 141)		2.22	1.70	.521
6	I feel capable of clearing an obstructed airway	Intervention (n = 149)	.240	3.17	2.55	.622
		Control (n = 141)		3.03	2.68	.345
7	I feel capable of performing adequate mask ventilation	Intervention (n = 149)	.181	2.89	1.81	1.082
		Control (n = 141)		2.86	1.95	.907
8	I feel capable of performing adequate chest compressions	Intervention (n = 149)	.181	2.04	1.50	.544
		Control (n = 141)		2.19	1.61	.538
9	I feel capable of using a defibrillator	Intervention (n = 149)	.066	2.54	1.81	.685
		Control (n = 141)		2.36	2.07	.286
10	I feel capable of distinguishing between a hyperdynamic and a hypodynamic cardiac arrest	Intervention (n = 149)	.016	3.68	1.42	2.257
		Control (n = 141)		3.38	1.64	1.738
11	If I encounter an unresponsive patient, I feel capable of performing Advanced Cardiac Life Support	Intervention (n = 149)	<.001	3.56	1.82	1.738
		Control (n = 141)		3.69	2.16	1.532

## Discussion

This randomized controlled simulation study explored the effectiveness of Peyton’s 4-step approach for ACLS training compared to the traditional 2-step method.^31^ There were no significant differences in conveying purely isolated tactile CPR skills, such as chest compressions or ventilation. However, the results do indicate that Peyton’s approach is more efficacious for the instruction of procedural CPR skills. Procedural skills are the overall performance of the ALS algorithm which also contribute to high-quality CPR by faster evaluation of cardiopulmonary arrest and overall reduced no-flow time.^11-14^

In contrast to our findings, a recent systematic review has highlighted inconclusive evidence regarding the effectiveness of Peyton's approach versus alternative stepwise methods in teaching CPR skills.^31^ This likely stems from the fact that the published studies analyze Peyton’s approach for different specific skill domains within CPR. Chest compressions,^38,39^ minimizing interruptions, and passing BLS^40^ or ATLS^41^ scenarios have been targeted as learning objectives but should not be compared directly. Each domain involves unique challenges and techniques, leading to variations in the outcomes measured. ACLS requires more procedural knowledge than BLS. Performing chest compressions demands less procedural knowledge than passing a BLS scenario. As a result, the low certainty evidence as previously described, may reflect these differences in focus, rather than providing a clear overall picture of which teaching method is superior.^31^

Furthermore, studies aiming to analyze the effectiveness of Peyton’s approach for ACLS are scarce and therefore lacking in the recent review.^31^

To provide a clearer insight to evaluate the effectiveness of the Peyton method, it should be analyzed for each CPR skill domain separately. This aligns with principles of learning psychology, which highlight that different learning objectives and competency levels require methods of instruction that are specifically adapted to the particular learning objective.^42,43^

A substantial number of published studies have demonstrated the effectiveness of the Peyton approach in enabling the acquisition of complex clinical competencies that are essential in an operative setting. These include competencies like the insertion of a central venous catheter (CVC), encompassing 39 treatment instructions.^24-27^

Although CPR has been identified as a less complex clinical skill,^44^ it is of critical importance, particularly in the context of ACLS, to differentiate between 2 main necessary skill domains.^45^ The first comprises fewer complex competencies, such as chest compressions. The second domain encompasses more intricate competencies, such as the effective execution of the algorithm which includes a sequence of actions after detecting cardiac arrest or irregular breathing.^32^ The differentiation of the ACLS skill domains can be aligned with the learning pyramid of Miller, which posits that clinical competencies are constituted by 4 hierarchical processes, consisting of following steps: (1) knowledge (theoretical knowledge of ACLS), (2) application of knowledge (ie, chest compressions), (3) clinical skills competency (applying the ACLS algorithm in a simulated setting), and (4) clinical performance (applying the ACLS algorithm in a real-life setting).^46,47^ Each process needs a specifically adapted instructional methods. In the context of ACLS, the Peyton approach offers several potential advantages by integrating a multitude of learning theories and instructional designs. Consequently, the approach is effective in targeting the various CPR skill domains through the application of its step-by-step methodology.^24,31,44,48^

The initial 2 steps are based on a section of the social learning theory and are referred to as “Model Learning.”^49,50^ Through these steps, fundamental haptic abilities (fewer complex skills), such as those required for chest compressions or bag-mask ventilation are conveyed.^44^ The actual skill complexity of ACLS lies within the time effective conduction of each separate skill in the correct sequence, which requires significant procedural knowledge.^45^ Consequently, a more profound level of learning, contextualization, and the capacity to transform theoretical knowledge into practical behaviors needs to be conveyed.^51^ This learning process is accelerated by the third step of the Peyton approach (learning by teaching), as it encourages the trainee to engage in a process of deep reflection and active recall of the learning content, thereby facilitating its retention to a greater extent.^48,52,53^ According to the principles of retrieval practice, the third step significantly enhances declarative memory.^54,55^ Moreover, it incorporates motor imagery, which facilitates a more profound encoding of the individual steps of the ACLS algorithm in comparison to mere observation (2-step approach).^48,56,57^ In line with these theoretical assumptions, the results of this study also confirm the deeper encoding of declarative memory through the Peyton approach, indirectly leading to better procedural competencies. The undergraduates of the Peyton group declared significant higher learning gains on the first item of the CSA “I feel capable of describing the algorithm of Basic Life Support or Advanced Cardiac Life Support,” which reflects improved declarative memory. As a result, the cognitive load was reduced, which in turn lead to an enhanced cognitive capability for the actual procedural performance.^58,59^ In concordance with this hypothesis, the undergraduates of the Peyton group did not only reach significant lower no-flow times and a better overall score of the CPR quality, but also reported significant higher learning gains on the items reflecting actual procedural knowledge of ACLS. Among other “If I encounter an unresponsive patient, I feel capable of performing ACLS.” Therefore, this study suggests the effectiveness of the Peyton approach by mainly enhancing the declarative memory of the single steps.

Similar findings were demonstrated in the context of gastric tube insertion, where procedural knowledge of undergraduates following Peyton’s approach was significantly higher. The colleagues also demonstrated superior NTS like professionalism and communication in the Peyton group.^24^ Nevertheless, the present study did not find any improvement of NTS in either group. A deeper insight into NTS in general and mainly during emergency care provides an explanation: improving NTS is detached from factual learning and requires far more reflection to adapt behavioral benchmarks into one's own behavior.^51^ The third step of Peyton may have accelerated this adaptation during nonstressful clinical tasks like a gastric tube insertion.^48^ Emergency simulation training leads to more stress than performing an isolated clinical task. It is known that emotional stress impedes NTS, therefore Peyton’s approach was unable to sufficiently convey NTS during for a simulated emergency setting.^60,61^ Both training methods of this study functioned as isolated instructional designs focusing more on TS. Furthermore, neither the Peyton approach, nor other instructional designs like the 2-step method are tailored to directly address behavioral patterns. Therefore, further didactic methods that target specifically NTS should be embedded within ACLS trainings to enhance overall CPR quality.^32^

Interestingly, most published studies exploring Peyton’s effectiveness for CPR mainly focus on the skills themselves, rather than considering the potential influence of trainees’ educational backgrounds and prior experience.^31^

In accordance with the learner-centered teaching approach,^62^ varying levels of education and experience (also familiarity with simulation settings) of the learners should be considered while implementing instructional strategies to facilitate the acquisition of skills.^24,30,40,41,63^ There is a paucity of evidence indicating the educational context in which the Peyton approach may be most suitable. To illustrate, when trainees are inexperienced with high-fidelity simulation, an elevated cognitive load may result in a subsequent decline in performance, thereby diminishing the potential advantages of the Peyton approach.^59^ Herein derives one strength of this study, which explored the Peyton approach in a predefined educational level. The participating undergraduates were familiar with high-fidelity simulation and had also passed BLS trainings. Therefore, the instruction effects could be analyzed with low bias, as the undergraduates were at low risk of cognitive overload.

A further strength of this study is that the TS were assessed using an observational method (Graham scoring) and a real-time feedback device, thereby integrating the strengths of both human judgment and technology. Research has demonstrated that real-time feedback enhanced CPR performance assessment^64^; however, there is still the need of human observational abilities for aspects that devices are unable to quantify, including awareness, breathing, circulation control, and the overall algorithm.^1^ Consequently, our findings enable the analysis of the efficacy of Peyton's approach on CPR quality with minimal bias.

## Limitations

One might argue that some endpoints of this study were assessed subjectively using scoring systems that were filled out by the instructors or by the undergraduates. Rating of the NTS has been conducted with the AS-NTS, which has been validated broadly, indicating a robust explanatory capacity.^17^

The CSA reflects indirectly the actual learning gain, as studies have shown good correlations of the perceived- and objective learning gain.^65^ Moreover, it is challenging to objectively assess enhanced declarative memory and even more so to quantify reduced cognitive load, which is indirectly reflected by the first item of the CSA: “I feel capable of performing the Advanced Life Support.” An alternative assessment method might have been a written test, which would have been mainly limited to declarative knowledge (eg, “I feel capable of describing the algorithm of Basic Life Support and Advanced Life Support”). Therefore, the CSA provides a more accurate reflection of the enhanced learning process, as it better captures procedural mastery.

Some additional factors may have contributed to potential limitations regarding the generalizability of our findings. The ACLS training of this study was conducted during the second year of medical school, an early stage in the medical curriculum. In contrast, many other medical schools implement ACLS training later in the educational trajectory, when students may possess more clinical knowledge and experience. As such, the participants in this study may have derived disproportionate benefit from the structured nature of the Peyton method due to their relative inexperience. Therefore, when interpreting and applying these results, differences in curriculum design and timing of ACLS instruction should be considered. Another source of potential bias stems from the instructors, who were not blinded to the teaching method. Their awareness of the instructional strategy being used might have inadvertently influenced their delivery, potentially leading to greater engagement or effort when teaching with the Peyton method compared to the 2-step approach.

## Conclusion

This study demonstrates that the Peyton method is particularly beneficial in stages of the medical curriculum when undergraduates have already accumulated foundational experience in resuscitation and emergency care. Compared to traditional 2-step methods, the Peyton method enhances the teaching of procedural CPR skills, improves adherence to the ACLS algorithm, and supports the acquisition and retention of procedural knowledge. These benefits translate into faster recognition of cardiopulmonary arrest, reduced no-flow times, and higher-quality CPR.

However, in the earlier stages of the medical curriculum, where learners are primarily developing discrete tactile skills such as chest compressions and ventilation, the Peyton method does not offer comparable advantages. These findings highlight the importance of strategically integrating the Peyton method at appropriate stages of medical education to maximize its educational impact.

## Supplemental Material

sj-docx-1-mde-10.1177_23821205251358090 - Supplemental material for The Effectiveness of Peyton’s 4-Step Approach to Teach Resuscitation Skills: A Randomized Controlled Clarification Study Supplemental material, sj-docx-1-mde-10.1177_23821205251358090 for The Effectiveness of Peyton’s 4-Step Approach to Teach Resuscitation Skills: A Randomized Controlled Clarification Study by Denisa Cenaj, Leonie Schulte-Uentrop, Leonie Fee Laura Kröger, Josephine Küllmei, Jan-Marcus Haus and Parisa Moll-Khosrawi in Journal of Medical Education and Curricular Development

sj-docx-2-mde-10.1177_23821205251358090 - Supplemental material for The Effectiveness of Peyton’s 4-Step Approach to Teach Resuscitation Skills: A Randomized Controlled Clarification Study Supplemental material, sj-docx-2-mde-10.1177_23821205251358090 for The Effectiveness of Peyton’s 4-Step Approach to Teach Resuscitation Skills: A Randomized Controlled Clarification Study by Denisa Cenaj, Leonie Schulte-Uentrop, Leonie Fee Laura Kröger, Josephine Küllmei, Jan-Marcus Haus and Parisa Moll-Khosrawi in Journal of Medical Education and Curricular Development
