# Supplementary material for: The Effectiveness of Peyton’s 4-Step Approach to Teach Resuscitation Skills: A Randomized Controlled Clarification Study
Source: J Med Educ Curric Dev. 2025 Jul 24;12:23821205251358090. doi: 10.1177/23821205251358090 (PMC12304607; doi:10.1177/23821205251358090)
Supplement: sj-docx-2-mde-10.1177_23821205251358090 - Supplemental material for The Effectiveness of Peyton’s 4-Step Approach to Teach Resuscitation Skills: A Randomized Controlled Clarification Study [file sj-docx-2-mde-10.1177_23821205251358090.docx]

Supplementary table 1. CPR metrics

| **CPR skill** | **Intervention group** | | **Control group** | | | |  |  |  | |
| --- | --- | --- | --- | --- | --- | --- | --- | --- | --- | --- |
|  | **MV** | **SD** | **MV** | **SD** | | | **T(df)** | **p** | **95% CI** |  |
|  |  |  |  |  | | |  |  | LL | UL |
| Compression: Correct rate (percentage) | 55.18 | 31.098 | 44.82 | | 30.278 | 1.860(120) | | .065 | -.667 | 21.388 |
| Compression: Correct release (percentage) | 52.88 | 28.505 | 59.74 | | 30.241 | -1.283(120) | | .202 | -17.464 | 3.729 |
| Compression: Hand position (correct percentage) | 66.09 | 34.145 | 66.32 | | 33.165 | -.037(120) | | .970 | -12.322 | 11.864 |
| Flow time (percentage) | 71.48 | 15.513 | 64.05 | | 21.845 | 2.132(120) | | .035 | .529 | 14.345 |
| Compression: Overall score | 37.68 | 28.958 | 38.24 | | 24.992 | -.115(120) | | .908 | -10.234 | 9.106 |
| Ventilation: overall score | 27.11 | 24.909 | 30.21 | | 28.036 | -.641(120) | | .523 | -12.691 | 6.481 |
| Compression: Hand position xiphoid error (percentage) | 33.91 | 34.145 | 29.05 | | 30.405 | .832(120) | | .407 | -6.708 | 16.439 |
| Compression: Correct depth (percentage) | 53.57 | 34.231 | 58.18 | | 31.352 | -.776(120) | | .439 | -16.374 | 7.153 |
| Maximum number of ventilations per cycle | 1.61 | 1.073 | 1.50 | | 1.140 | .531(120) | | .596 | -.292 | ,506 |
| Average number of compressions per cycle | 39.29 | 20.475 | 29.50 | | 12.727 | 3.212(119) | | .002 | 3.756 | 15.826 |
| Max number of compressions per cycle | 90.86 | 57.662 | 56.41 | | 30.656 | 4.205(120) | | <.001 | 18.229 | 50.667 |
| Time to first compression | 18.79 | 19.336 | 34.00 | | 39.654 | -2.61(120) | | .010 | -26.720 | -3.708 |
| Time to first ventilation | 50.30 | 50.205 | 79.05 | | 90.128 | -2.12(120) | | .036 | -55.553 | -1.931 |
| Compression: Correct release (percentage) | 52.88 | 28.505 | 59.74 | | 30.241 | -1.28(120) | | .202 | -17.464 | 3.729 |
| Compression: Hand position correct (percentage) | 66.09 | 34.145 | 66.32 | | 33.165 | -.037(120) | | .970 | -12.322 | 11.864 |
| Compression: Mean depth | 4904.91 | 970.855 | 4839.17 | | 1293.422 | .313(120) | | .755 | -350.379 | 481.867 |
| Compression: Mean release depth | 491.79 | 193.005 | 398.00 | | 246.831 | 2.307(120) | | .023 | 13.290 | 174.698 |

Abbreviations: M = Mean value; SD = Standard deviation; T = t-distribution; df = Degrees of freedom; CI = confidence interval; LL = lower limit; UL = upper limit.
